# Association of Vitamin D Deficiency With Glycemic Control in Type 2 Diabetes Mellitus

**DOI:** 10.7759/cureus.92179

**Published:** 2025-09-12

**Authors:** Hafiz Muhammad Faizan Mughal, Kashaf Ali, Bhavna Singla, Shivam Singla

**Affiliations:** 1 Internal Medicine, Khawaja Muhammad Safdar Medical College, Sialkot, PAK; 2 Pathology, Ziauddin Hospital, Karachi, PAK; 3 Internal Medicine, Erie County Medical Center (ECMC), Buffalo, USA; 4 Internal Medicine, TidalHealth Penninsula Regional, Salisbury, USA

**Keywords:** 25-hydroxyvitamin d 2, diabetes mellitus, glycated hemoglobin, insulin resistance, type 2, vitamin d deficiency

## Abstract

Background

Vitamin D is widely acknowledged for its important role in sugar metabolism by influencing inflammatory control, insulin production, and sensitivity. One of the main causes of problems in individuals with type 2 diabetes mellitus (T2DM) is inadequate glycemic management. This study aimed to evaluate the association between glycemic management and vitamin D levels in individuals with T2DM.

Methods

A total of 200 adult T2DM patients participated in this cross-sectional observational study at the endocrinology department in a tertiary care hospital as outpatients. The participants were divided into two subcategories based on blood glucose control: with a good HbA1c (<7%; n = 76) and with a poor HbA1c (≥7%; n = 124). To measure the levels of serum 25-hydrovitamin D (25(OH)D), a chemiluminescence immunoassay was utilized. 25(OH)D < 20 ng/mL was the indication for vitamin D insufficiency. BMI, gender, age, diabetes duration, and medication schedule were among the demographic and clinical data collected. The statistical analysis employed the chi-square test, the independent t-test, and the Pearson correlation. Statistical significance was defined at p-value <0.05.

Results

Vitamin D deficiency was present in 145 (72.5%) participants, and 101 (81.5%) individuals demonstrated poor glucose management compared to 44 (57.9%) participants in the good control group (p < 0.001). Patients with poorly controlled diabetes had substantially lower mean 25(OH)D levels (15.8 ± 5.9 ng/mL) than those with well-controlled diabetes (22.1 ± 6.3 ng/mL; p < 0.001). There was a negative correlation (r = -0.45; p < 0.001) between vitamin D levels and HbA1c.

Conclusion

In patients with T2DM, inadequate glycemic management is associated with vitamin D insufficiency. The findings suggest that, in the context of comprehensive diabetes care, monitoring and adjusting vitamin D levels are beneficial.

## Introduction

Type 2 diabetes mellitus (T2DM) is a major world health problem, marked by ineffective pancreatic beta-cell response, insulin insensitivity, and chronically elevated glucose concentration in the blood. It affects over 537 million adults worldwide, with numbers expected to increase significantly in the next decade [1]. Glycemic control is important for diabetes management, as it reduces the risk of macrovascular and microvascular complications. Nevertheless, despite therapeutic advances, many patients remain inadequately controlled [2].

Vitamin D, a fat-soluble secosteroid, is primarily known for its role in bone health, but it has gained attention in glucose metabolism and insulin signaling [3]. The active metabolite 1,25-dihydroxyvitamin D (25(OH)D) activates vitamin D receptors in pancreatic β-cells and peripheral tissues, affecting insulin synthesis, secretion, and sensitivity [4]. Moreover, it has immunomodulatory and anti-inflammatory effects, relevant to T2DM pathogenesis [5].

Increased Hb1Ac, difficulty in glucose intolerance, and insulin resistance are associated with low levels of 25(OH)D in serum. Hypovitaminosis D is common in patients with diabetes, indicating a possible association between low vitamin D levels and poor glycemic control [6]. Nevertheless, the results of interventional studies are not conclusive due to the heterogeneity of the baseline vitamin D status, dose of interventions, and follow-up periods [7]. Many studies have not adjusted for confounding variables, including BMI, disease duration, and insulin use. It is crucial to clarify this relationship because vitamin D is a modifiable factor that may supplement the current approach to diabetes care [8].

Investigating the correlation between blood vitamin D levels and glycemic control in T2DM patients was the objective of this study. It evaluated the association between elevated Hb1Ac and vitamin D deficiency. Furthermore, the purpose was to identify a modifiable factor to improve diabetic care.

## Materials and methods

This study was conducted at the Outpatient Endocrinology Department of a tertiary care facility from October 2023 to March 2024 (Ref: 4441MS/21) via a consecutive sampling technique. The primary purpose was to determine the association between blood vitamin D levels and glycemic control in adults with T2DM.

A consecutive non-probability sampling technique was utilized to recruit adult patients on regular follow-up visits after obtaining informed written consent. The sample size of 200 was calculated based on a 95% confidence level, 5% margin of error, and 70% expected prevalence of vitamin D deficiency, using OpenEpi version 3.0.0 (released 2013, Dean AG, Sullivan KM, Soe MM, Emory University, Atlanta, GA) [9].

The adult population aged ≥30 years with a proven T2DM diagnosis for at least one year, and those who were scheduled to receive outpatient follow-up and were willing to undergo blood testing for 1,25(OH)D and HbA1c, and provide informed consent, were eligible to participate. Exclusion criteria included type 1 or gestational diabetes, renal or liver impairment, recent intake (last three months) of vitamin D, interfering drugs or supplements (glucocorticoids, anticonvulsants), malabsorption disorder (diarrheal or inflammatory bowel dysfunction), and cognitive or mental illness, pregnancy, and lactation.

The sample was categorized into two groups based on glycemic control (<7% and ≥7% HbA1c). Instead of active intervention, natural exposure to vitamin D deficiency was measured. Study variables included the length of diabetes, medication history (insulin and metformin), comorbidities, blood 25(OH)D levels, and demographics, including age, gender, and BMI. Venous blood samples (5 mL) were drawn in the morning (8-10 am) at the antecubital vein following an eight- to 12-hour overnight fast. Serum vitamin D and HbA1c levels were measured using the chemiluminescence immunoassay and HPLC, respectively. There was no external control for adherence to exposure. SPSS version 26.0 (IBM Corp., Armonk, NY) was used; independent t-tests were used for continuous variables, and chi-square tests were utilized to compare categorical variables between groups (p < 0.05).

## Results

Two hundred patients with T2DM were evaluated in this study to determine the correlation between vitamin D levels and glycemic control. Patients with inadequate glycemic management had substantially higher vitamin D inadequacy. An inverse association was demonstrated between vitamin D concentration and sugar control. These findings imply that vitamin D supplements may help diabetics control their blood sugar levels. Table 1 illustrates the clinical and demographic features of research subjects with T2DM.

**Table 1:** Study population’s clinical and demographic features BMI: basal metabolic index; n: number of participants; SD: standard deviation; T2DM: type 2 diabetes mellitus; %: percentage; *: significance at p-value <0.05

Parameter	Total (n = 200)	<7% HbA1c glycemic control (n = 76)	≥7% HbA1c glycemic control (n = 124)	Statistical test	Test value	p-value
Age (years) (mean ± SD)	54.3 ± 9.6	53.1 ± 9.2	55.2 ± 9.9	t-test	1.46	0.146
Male sex (%)	114 (57.0%)	44 (57.9%)	70 (56.5%)	Chi-square	0.03	0.860
BMI (kg/m²) (mean ± SD)	28.6 ± 4.2	27.8 ± 4.1	29.1 ± 4.3	t-test	2.02	0.045*
Duration of T2DM (years)	8.1 ± 5.6	6.7 ± 5.0	9.0 ± 5.8	t-test	2.95	0.004*
Insulin use (%)	76 (38.0%)	18 (23.7%)	58 (46.8%)	Chi-square	10.07	0.002*
Metformin use (%)	162 (81.0%)	64 (84.2%)	98 (79.0%)	Chi-square	0.78	0.376

In patients with glycemic control with ≥7% HbA1c, duration of diabetes, use of insulin, and BMI were profoundly higher than those of individuals with <7% HbA1c(p = 0.002, p = 0.004, p = 0.045, respectively). No major disparities based on age, sex, and use of metformin were observed. These findings suggest that obesity, prolonged disease duration, and insulin treatment worsen the glycemic status among T2DM patients. Table 2 indicates the vitamin D status in groups with <7% and ≥7% HbA1c.

**Table 2:** Vitamin D status and glycemic control 25(OH)D: 25-hydroxyvitamin D; n: number of participants; SD: standard deviation; %: percentage; *: significance at p-value <0.05

Variable	Total (n = 200)	<7% % HbA1c glycemic control (n = 76)	≥7% HbA1c poor glycemic control (n = 124)	Statistical test	Test value	p-value
Serum 25(OH)D (ng/mL) (mean ± SD)	18.5 ± 6.7	22.1 ± 6.3	15.8 ± 5.9	t-test	7.09	<0.001*
Vitamin D deficient (%) (<20 ng/mL)	145 (72.5%)	44 (57.9%)	101 (81.5%)	Chi-square	13.06	<0.001*
Vitamin D insufficient (20-29 ng/mL) (%)	35 (17.5%)	21 (27.6%)	14 (11.3%)	Chi-square	8.89	0.003*
Vitamin D sufficient (≥30 ng/mL) (%)	20 (10.0%)	11 (14.5%)	9 (7.3%)	Chi-square	2.69	0.101

Patients with inadequate glycemic management had a significantly lower average blood 25 (OH)D (15.8 ± 5.9; p < 0.001). 101 (81.5%) of patients with inadequate glycemic management reported vitamin D deficiency (<20 ng/mL), compared to 44 (57.9%) in the <7% HbA1c group (p < 0.001). These results highlight that poor glycemic management and lower vitamin D concentration are associated. Correlation between Hb1Ac and concentration of vitamin D is presented in Table 3.

**Table 3:** Correlation between Hb1Ac and vitamin D levels 25(OH)D: 25-hydroxyvitamin D; BMI: basal metabolic index; HbA1c: hemoglobin A1c; T2DM: type 2 diabetes mellitus; %: percentage; *: significance at p-value <0.05

Variable pair	Correlation coefficient (r)	Statistical test	Test value (t)	p-value
HbA1c (%) vs. 25(OH)D (ng/mL)	-0.45	Pearson’s r	-7.23	<0.001*
BMI vs. 25(OH)D	-0.18	Pearson’s r	-2.57	0.011*
Duration of diabetes vs. 25(OH)D	-0.21	Pearson’s r	-3.05	0.003*

The level of HbA1c and 25(OH)D had a strong negative correlation (r = -0.45, p < 0.001), suggesting that higher HbA1c status was linked to low concentration of vitamin D. Additionally, there was a weak negative association with both diabetes duration (r = -0.21, p = 0.003) and BMI (r = -0.18, p < 0.011), indicating that insufficient vitamin D may have a modest association with disease duration and BMI and T2DM.

## Discussion

Investigating the relationship between inadequate vitamin D and insufficient glycemic management in individuals with T2DM was the study’s aim. The findings indicated that 25(OH)D and HbA1c had a significant inverse relationship. According to these results, low vitamin D is associated with poor glycemic management and may be a factor in the overall metabolic balance deterioration in diabetes patients.

Poor glycemic control was associated with a higher rate of vitamin D deficiency and a reduced mean level of serum 25(OH)D. These results align with the known studies indicating that low levels of vitamin D are associated with an increase in HbA1c levels [10]. Another study found that mild to moderate vitamin D deficiency was also linked to poor insulin sensitivity, which demonstrates the metabolic significance of low vitamin D status [11]. These effects are suggested to be mediated by alteration of the insulin receptor expression and glucose transporter activity in adipose and muscle tissue [12].

Additionally, this study discovered a weak but inverse relation between vitamin D levels and both diabetes duration and BMI. This trend is explained by data showing that in individuals with obesity, vitamin D is biologically less available, as it is sequestered in adipose tissue [13]. Chronic hyperglycemia has been shown to inhibit renal hydroxylation of vitamin D, which may explain the reduced levels in patients with prolonged disease [14].

Although vitamin D supplementation has been shown to have no significant effect on glycemic control in some clinical trials, others have demonstrated that correction of vitamin D deficiency is associated with improved insulin resistance [15]. It has also been indicated that postprandial glucose concentrations are reduced after supplementation with large amounts of vitamin D in deficient individuals [16]. The disparities in results may stem from the differences in population characteristics, vitamin D dosing schemes, and base scaling [17]. However, variation in the genetic expression of vitamin D receptors may be one of the factors in determining response to treatment [18]. Nevertheless, although inconsistent, vitamin D is under investigation as a biologically modifiable and low-cost factor of glycemic control [19].

The limitations that preclude concluding causality were sunlight exposure, dietary pattern, physical activity, and seasonal variation, which were not measured as additional confounding variables. A single-center and outpatient population reduces the extent of generalizability. Future studies should emphasize longitudinal and randomized experiments to determine causality and assess the vitamin D optimization on long-term metabolic outcomes.

## Conclusions

A significant correlation between poor glycemic management and lower concentrations of vitamin D in T2DM was established in this study. It was observed that there was an inverse relationship between the incidence of vitamin D deficiency and lower 25(OH)D in diabetic individuals with poor glycemic regulation. These results support the purpose of the study by demonstrating that a deficiency in vitamin D is associated with high levels of HbA1c and can play a role in the development of disorders related to glucose control.

According to these results, vitamin D concentration may be a controllable element in diabetes treatment. Including regular vitamin D screening and specific treatment can contribute to improved metabolic performance and should be adopted as an economical addition to current glycemic controls.
